# A Manual of Physiology, Including Physiological Anatomy, for the Use of the Medical Student

**Published:** 1846-07

**Authors:** 


A Manual of Physiology, including Physiological Anatomy, for the Use of the Medical Student. By *William B. Carpenter,* M.D. F.R.S. With One Hundred and Eighty Illustrations. 12mo. pp. 582. London: Churchill, 1846.

The present volume combines in a condensed form, the matter contained in the two larger works of Dr. Carpenter on Vegetable, Comparative, and Human Physiology; it thus presents an outline of the vital phenomena, as they have been investigated up to the present time. Although this manual combines in some degree the scope of the above works, yet the author informs us, it cannot be regarded as a mere abridgement of them. As we have already noticed at some length, and with much approbation, Dr. Carpenter's Principles of Physiology (see Medico-Chirurgical Review, 1845, p. 321), our remarks on the present occasion will be limited.

The first book, treating of General Physiology, contains a judicious sketch of the *nature* and *objects* of this science, which we would commend to the careful consideration of our junior brethren, with the conviction, that it is a matter of the first consequence, to obtain a just conception of the fundamental laws, and of the scope of the inquiry in the prosecution of every branch of knowledge, but in a more especial degree as regards the most abstruse of all sciences—that of life. To him who, neglecting the natural order, commences his investigation with the individual actions of vitality, infinitely varied and complex as they are, all appears peculiar, bizarre, and obscure; and if he is satisfied to be guided by those writers who, setting aside as unconcerned with human physiology, the general laws of matter, of chemical affinity, of vegetable organization, and of comparative anatomy, he will rest in the false assumption that, living bodies in their structure and functions, form an exception to the ordinary phenomena of Nature. The student of physiology is particularly prone to fall into one error which has often led to the most mischievous results; it is this—the belief that more is known of the phenomena and essence of inorganic matter, than of those connected with organized bodies. One of the evils of this misconception has been the attempt, often made and always in vain, to determine that "ultimate fact" of organization, the essential nature of life. Well would it have been for the progress of our science, if physiologists, in place of these barren speculations, recollecting that we only know matter, whether inorganic or organic, by its qualities, had learnt to recognise that line of demarcation, which, separating natural phenomena from their efficient causes, has never yet been passed by the most eager or the profound of human observers.

Entertaining these convictions, we have perused with much satisfaction the following remarks of Dr. Carpenter.

"All sciences have their 'ultimate facts;' that is, facts for which no other cause can be assigned than the Will of the Creator. Thus, in physics, we cannot ascend above the fact of Attraction (which operates according to a simple and universal law) between all masses of matter; and in chemistry, we cannot rise beyond the fact of Affinity (limited by certain conditions which are not yet well understood) between the particles of different kinds of matter. When we say that we have *explained* any phenomenon, we merely imply that we have traced its origin to these properties, and shown that it is a necessary result of the laws according to which they operate. For the existence of the properties, and the determination of the conditions, we can give no other reason than that the Creator *willed* them so to be; and, in looking at the vast variety of phenomena to which they give rise, we cannot avoid being struck with the general harmony that exists amongst them, and the mutual dependence and adaptation that may be traced between them, when they are considered as portions of the general economy of Nature.—There is no difference in this respect between Physiology and other sciences; except that the number of these (apparently) ultimate facts is at present greater in physiology than it is in other sciences, because we are not at present able to include them under any more general expression. Thus we find a certain peculiar endowment existing in one form of structure; and another endowment, equally peculiar, inherent in another; but we can give no reason why the structure called muscular, should possess contractility, and why the structure called nervous should be capable of generating and conveying the force which excites that contractility to action. Each of these facts, therefore, is for the present the limit to our knowledge; we can ascertain the conditions, according to which the muscular contractility, and the exciting power of the nerve, are called into operation, and can form some estimate of the amount of the forces which they generate; but we cannot see clearly that they are necessarily connected by any common tie, such as that which binds together the planetary masses, at the same time that it weighs down the bodies on the surface of the earth towards its centre." P. 15.

Another of the fallacies which still lingers in the Schools, and which, as we have said, is traceable to the too exclusive study of vital actions, is the notion that these phenomena are altogether sui generis; whilst the truth is, that the processes of the vegetative existence especially, are to a great extent dependent upon the ordinary energies of matter.

"There can be no doubt whatever," observes the author, "that, of the many changes which take place during the *life*, or state of *vital activity*, of an Organized being, and which intervene between its first development and its final decay, a large proportion are effected by the agency of those forces, which operate in the Inorganic world; and there is no necessity whatever for the supposition, that these forces have any other operation *in* the living body than they would have *out* of it under similar circumstances. Thus the propulsion of the blood by the heart through the large vessels, is a phenomenon precisely analogous to the propulsion of any other liquid through a system of pipes by means of a forcing pump; and if the arrangement of the tubes, the elasticity of their walls, the contractile power of the heart, and the physical properties of the fluid, could be precisely imitated, the artificial apparatus would give us an exact representation of the actions of the real one. The motor force of the muscles upon the bones, again, operates in a mode that might be precisely represented by an arrangement of cords and levers; the peculiarity here, as in the former case, being solely in the mode in which the force is first generated. So, again, the digestive operations which take place in the stomach are capable of being closely imitated in the laboratory of the chemist; when the same solvent fluid is employed, and the same agencies of heat, motion, &c. are brought into play. Moreover we shall hereafter see reason to believe, that the peculiar form of Capillary Attraction, to which the term Endosmose is applied, performs an important part in the changes which are continually taking place in the living body." P. 10.

The recognition of the close resemblance, and not unfrequently of the actual identity, thus displayed in the processes of the vegetative life, when compared with the ordinary phenomena of physics and chemistry, naturally suggests the inquiry how far it may be possible to refer *all* the actions of life to properties of matter. In discussing such a question one thing is immediately apparent, namely, that with our present knowledge, we can only argue from analogy and by inference, inasmuch as the *known* properties of matter are altogether insufficient to produce, for example, muscular contraction and nervous power. Dr. Carpenter has a very interesting section "on the Connexion between Vitality and Organization," which should be read throughout to realise the scope of the argument, but we will endeavour to convey in our limited space the views set forth upon this profoundly important inquiry. The author commences by observing that—

"The idea that new properties may be called forth or developed by a new combination of elements, and by a new arrangement of particles,—and that, consequently, the class of properties included under the general term *vital* is dependent upon the peculiar state of matter which is designated as *organized,*—is so perfectly conformable to what is seen elsewhere, and is so fully sufficient to explain all observed phenomena, that it would scarcely seem necessary to use any further argument in support of it. But the notion has been entertained, that Vitality is *a something superadded* to matter, and that it is absurd to suppose that the phenomena of Life, can be produced by *any* combinations of matter; and this indeed so generally prevails, that it seems desirable to carry our investigations with regard to the causes of Vital phenomena a little further." P. 33.

Dr. Carpenter, then, shows that the properties of any kind of matter, even those with which we are most familiar, require *certain conditions* for their manifestation; that these properties may be either lateut or active, according as these conditions are or are not present; that, for example, oxygen and hydrogen have within themselves the latent property of so combining together as to form water; but this does not manifest itself so long as they are separate; nor does it manifest itself at ordinary temperatures, when they are mingled together; but if through such a mixture we transmit an electric spark, or if we raise the temperature by the contact of a heated body, or if we simply introduce into it a portion of platinum in a state of minute division, the requisite stimulus or excitation is given, and chemical union of the two substances is the result.

" Now if we apply these views to the phenomena of Life and Organization, we see that they enable us to regard those phenomena as *analogous* in character to those of the Inorganic world, though not *identical* with them; and they lead to a simplification of our ideas of them, which more clearly marks out the path to be pursued in their investigation. We find that the essential materials of Animal and Vegetable structures are the four elements, Oxygen, Hydrogen, Carbon, and Nitrogen; these are distinguished by the extraordinary number and variety of the combinations into which they will enter,—so much so, indeed, as to constitute, in this respect, a group quite distinct from all the other elementary substances. Now we are perfectly justified by what we elsewhere see, in attributing to these elements the property or dormant capability of exhibiting *vital* actions (in addition to the ordinary chemical ones with which we are familiar), so soon as they are placed in the requisite conditions; in other words, as soon as they are made a part of the living system by the process of Organization. It is only the peculiarity of the conditions required to manifest this capability, which prevents us from recognizing it as an ordinary property of matter, or at least of those forms of it, which we know by experience to be capable of entering into organized structures." P. 36.

" The elements, Oxygen, Hydrogen, Carbon, and Nitrogen, in a certain state of combination and arrangement, form the substance which we term Muscular fibre; and they then manifest certain peculiar properties, which we designate as *vital*. On the other hand, those same elements exist in nearly the same proportions, but in a different state of combination and arrangement, in the substance which we term Cyanate of Ammonia; and they then exhibit a different set of properties, which we call Physical and Chemical. Now we have just as much right to say, that the contractility of muscular fibre results from the peculiar combination and arrangement of the elementary particles in its substance, as we have to say that the solidity, translucency, hardness, and other qualities of the salt (all of which are opposed to the vital properties, and cannot co-exist with them,) are necessarily connected with *its* peculiar mode of combination and crystalline aggregation. If we were *only* acquainted with these elements as they exist in organic compounds, their transposition into a crystalline salt would be almost as marvellous to us, as the opposite change is now." P. 37.

The author, in considering the opposite theory, or that according to which Vitality is regarded as something distinct from and superadded to organized structure, opposes to it the facts disclosed by embryology.

He contends that it seems absurd to suppose that, in a single cell-germ, a molecule almost invisible even with a high magnifying power, a force is concentrated, which is afterwards to be diffused through the whole structure of a vast tree, or through the ever-changing fabric of a complex animal. On the other hand, it is affirmed, that we get rid of every difficulty, whilst at the same time we reason in accordance with the fundamental principles of Logic and Philosophy, which forbid us to assume any agency that is not required to explain the phenomena, if we suppose that Vital properties are called forth or developed in the substance of the germ, whilst this substance is being organized by the agency of its parent; that the germ, in its turn, calls forth or excites the dormant properties of the combining elements (like the spongy platinum, or the electric spark in a mixture of oxygen and hydrogen); that it thus originates, first a chemical combination, and then a peculiar structural arrangement, the elements then manifesting peculiar properties in place of their old ones, which last as long as they exist in that condition ; and so on.

Such, briefly, is the theory advocated by Dr. Carpenter in the work before us ; that it is characterized by the talent and extensive acquirements of its author, our readers will have themselves perceived. But, ingenious and even probable as we admit these views to be, it is necessary to point out that they are not, and, indeed, for the reasons already stated, in the present state of our knowledge, cannot be proved : to do that it would be necessary that we should know all the properties of matter, and the conditions necessary to call them forth into active exercise. In the interval, vast as it probably will be, which will elapse ere this information be attained, we hold that the subject is one fairly belonging to physiological research : and when it is discussed in the spirit which is alone befitting such inquiries, with sentiments that is to say of profound reverence towards the Deity, and with entire faith in the Christian revelation, no evils can result from the attempt to solve one of the most deeply interesting truths connected with our present state of being.

In the chapter on Vital Stimuli, the reader will find a large mass of interesting matter relating to the influence more especially of light, heat, and electricity upon the vegetable and animal kingdoms. This is an inquiry of the deepest importance both in physiology and pathology ; and which, notwithstanding the attention that has been directed to it of late years, particularly since the appearance of Dr. Edwards's admirable rearches upon the influencc of Physical Agents on Life, is still not sufficiently appreciated by practitioners. The effects produced by light have been principally investigated in plants ; but, as the author observes, there is abundant proof that this agent exercises an important influence on the nutritive processes of animals : thus the appearance of animalcules in infusions of decaying organic matter is much retarded, if the vessel be altogether secluded from light ; and it has been ascertained that, if an equal number of silk-worms' eggs be preserved in a dark room, and be exposed to common day-light, a much larger proportion of larvæ are hatched from the latter than from the former. It may be further remarked that—

"Numerous facts, collected from different sources, lead to the belief that the healthy development of the Human body, and the rapidity of its recovery from disease, are greatly influenced by the amount of light to which it has been exposed. It has been observed, on the one hand, that a remarkable freedom from deformity exists amongst nations who wear very little clothing; whilst, on the other, it appears certain that an unusual tendency to deformity is to be found among persons brought up in cellars or mines, or in dark and narrow streets. Part of this difference is doubtless owing to the relative purity of the atmosphere in the former case, and the want of ventilation in the latter ; but other instances might be quoted, in which a marked variation presented itself, under circumstances otherwise the same. Thus, it has been stated by Sir A. Wylie (who was long at the head of the medical staff in the Russian army), that the cases of disease on the dark side of an extensive barrack at St. Petersburgh, have been uniformly, for many years, in the proportion of three to one, to those on the side exposed to strong light. And in one of the London Hospitals, with a long range of frontage looking nearly due north and south, it has been observed that residence in the south wards is much more conducive to the welfare of the patients, than in those on the north side of the building." P. 56.

The vast influence exerted by heat, as a stimulus to vital action, is constantly exhibited in the two organic kingdoms ; and, without regarding its operation on a grand scale as displayed by contrasting the progress of vegetation in the tropical and polar regions of the earth, we may often, by observing some of the individual actions of life, obtain a striking illustration of the direct effects produced by this all-pervading and mysterious agent. The process of incubation affords such an example : here the elevation of temperature directly excites the previously dormant powers of the germ into activity, and throughout the whole process of development, sustains the actions essential to the formation of the chick. Again, if the egg be opened subsequently to the completion of the area vasculosa on the fourth day, and be carefully transferred into lukewarm water, and the actions of the heart be watched, it will be found that in a short time the organ beats more slowly and feebly, but that, on again raising the temperature of the water, the contractions become immediately quickened and in the ratio of the heat applied ; in this way the stimulating influence of heat is brought directly under the observation of the senses. The following are a few of the many details adduced on this subject by the author. As examples of the adaptation of vegetables to extremes of temperature, it is found that in a hot spring in the Manilla islands, of the temperature of 187°, plants flourish ; in one of the hot springs of Iceland, which boiled an egg in four minutes, a species of Chara was found growing ; various Confervæ, &c. have been observed in the boiling springs of Arabia and the Cape of Good Hope ; and at the island of New Amsterdam, there is a mud-spring, which, though hotter than boiling-water, gives rise to a species of Liverwort. On the other hand there, is the Lichen, serving as the winter food of the Rein-deer, which spreads itself over the ground whilst thickly covered with snow ; and the beautiful little Protococcus Nivalis, or Red Snow, reddens extensive tracts in the arctic regions, where the perpetual frost of the surface scarcely yields to the influence of the solar rays at midsummer.

We have only space for one more extract relating to a curious point in the economy of the bee.

"In order to hasten the development of the pupæ of the Social Bees, a very curious provision is made. There is a certain set, to which the name of Nurse-bees has been given, whose duty it is to cluster over the cells in which the Nymphs or Pupæ are lying, and to communicate the heat to them, which is developed by the energetic movements of their own bodies, and especially by respiratory actions of extreme rapidity. The nurse-bees begin to crowd upon the cells of the nymphs, about ten or twelve hours before these last come forth as perfect Bees. The incubation (for so it may be called) is very assiduously persevered in during this period by the Nurse-bees; when one quits the cell, another takes its place; and the rapidity of the respiratory movements increases, until they rise to 130 or 140 per minute, so as to generate the greatest amount of heat just before the young bees are liberated from the combs. In one instance, the thermometer introduced among seven nursing-bees stood at 92½° ; the temperature of the external air being 70°. We observe in this curious propensity a manifest provision for accelerating the development of the perfect Insect, which requires (as already pointed out) a higher temperature than the larva, in virtue of its greater activity. The Nurse-bees do not station themselves over the cells which are occupied by the larvæ; nor do they incubate the nymph-cells with any degree of constancy and regularity, until the process of development is approaching its highest point." P. 74.

In our present notice, we have confined ourselves to the introductory portion of Dr. Carpenter's Manual, which contains, mixed with many details of interest, an excellent sketch of General Physiology. The whole work is executed in the same spirit, and is brought up to the level of the day, all the important facts which have lately been added to our stock of knowledge being incorporated in the several sections. There are two plates and not less than 153 well executed wood-cuts dispersed through the work, which we have much pleasure in commending to the favourable notice of our readers.

